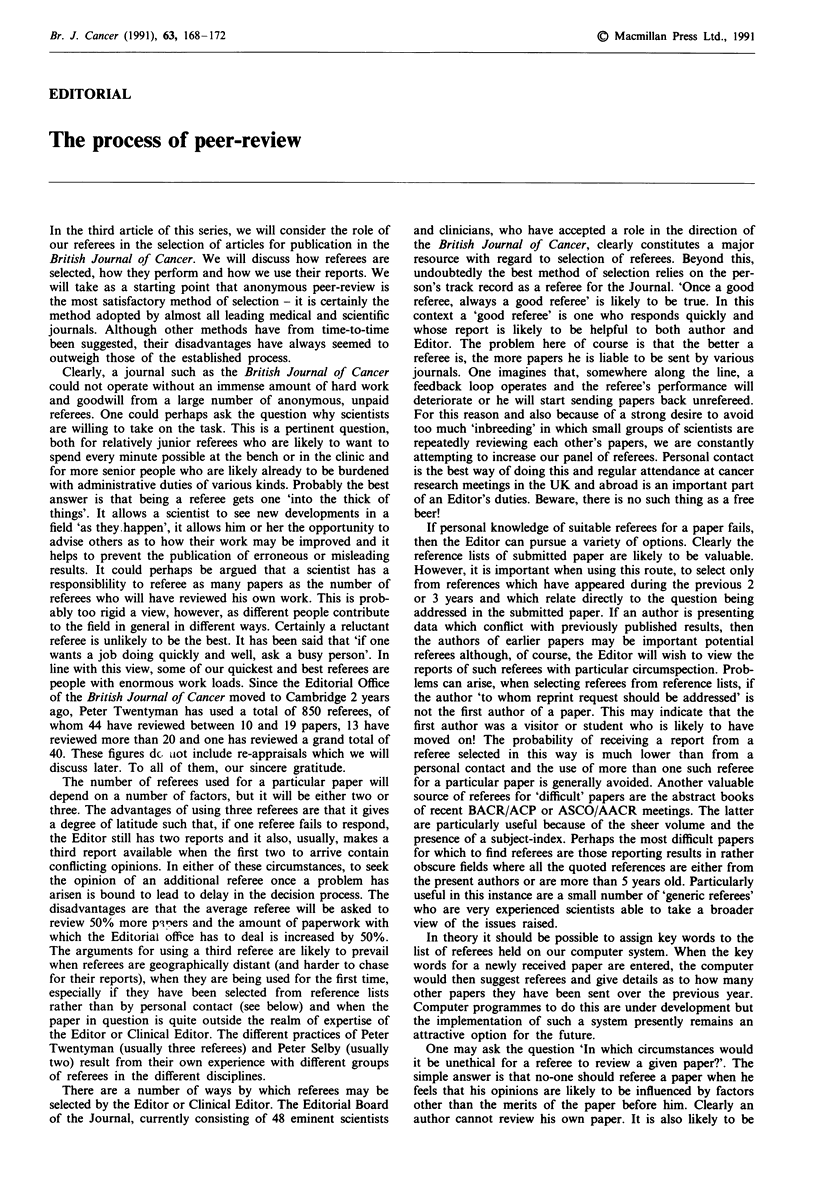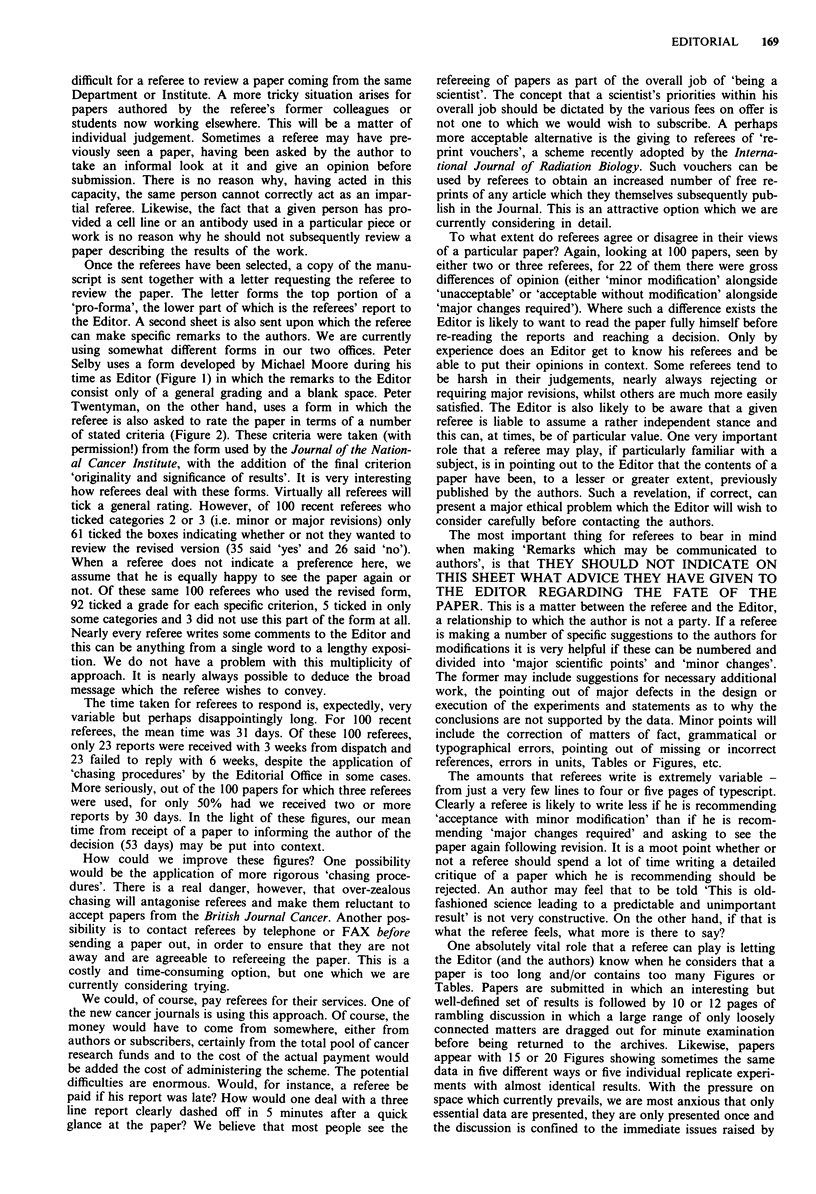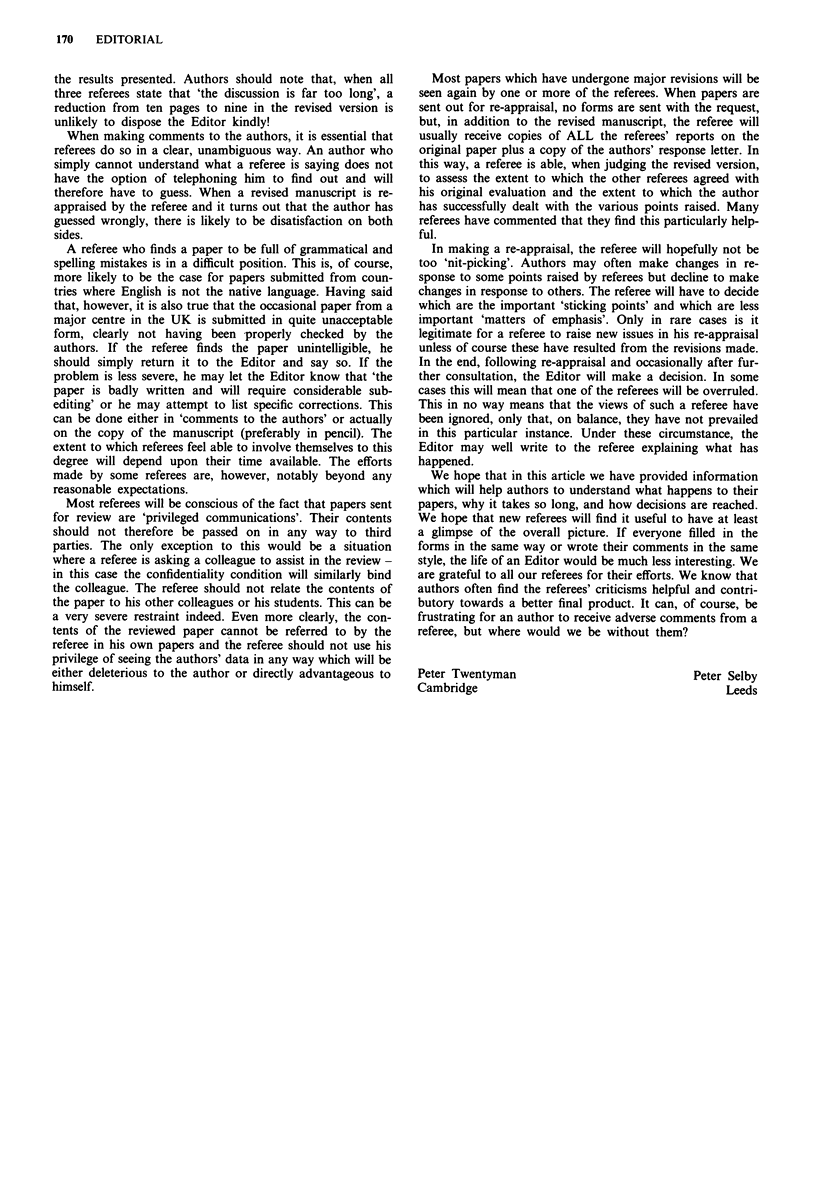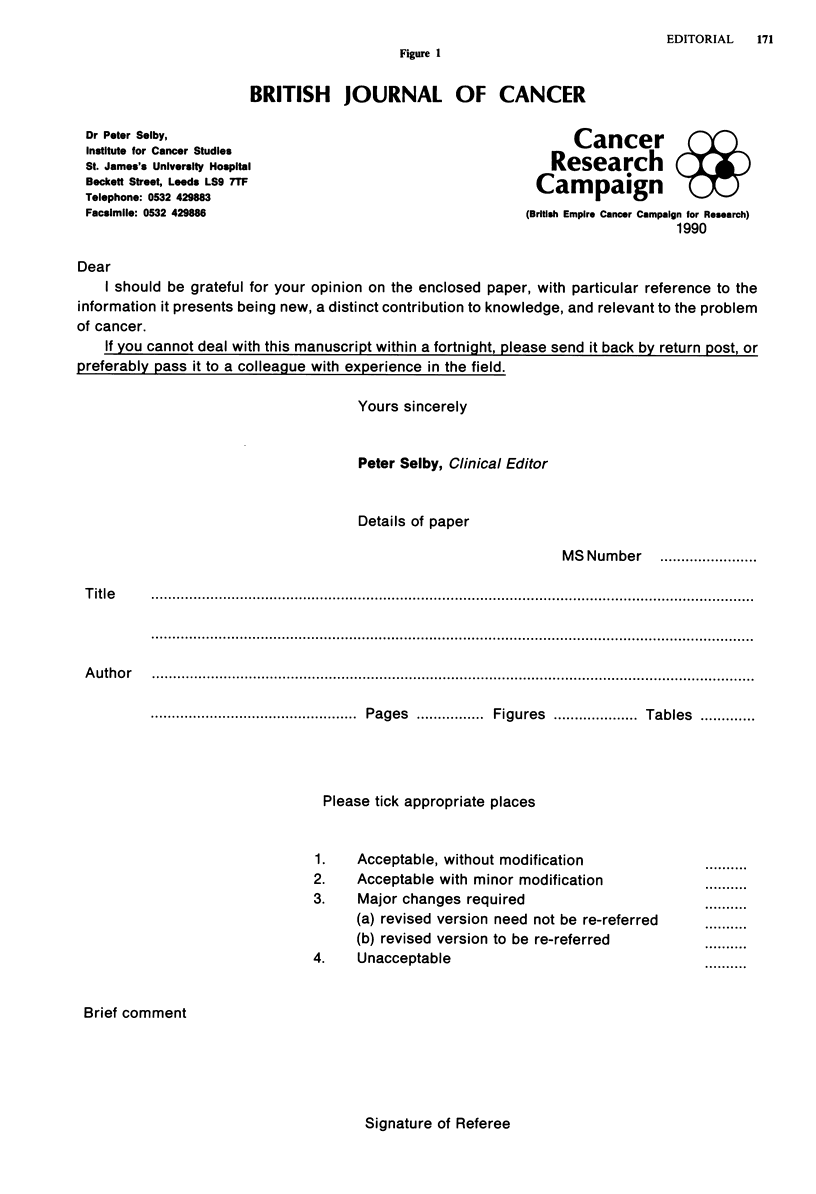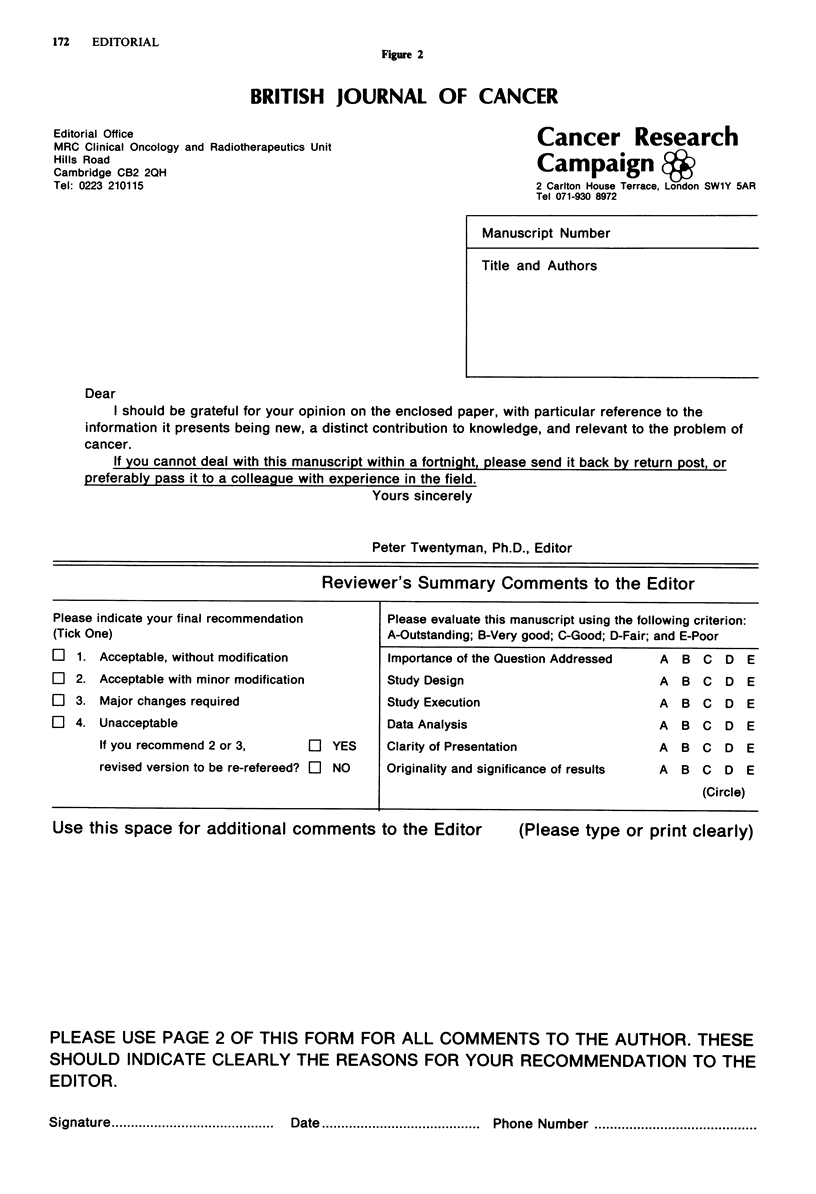# The process of peer-review.

**DOI:** 10.1038/bjc.1991.41

**Published:** 1991-02

**Authors:** P. Twentyman, P. Selby


© Macmillan Press Ltd., 1991

EDITORIAL

### The process of peer-review

In the third article of this series, we will consider the role of our referees in the selection of articles for publication in the *British Journal of Cancer*. We will discuss how referees are selected, how they perform and how we use their reports. We will take as a starting point that anonymous peer-review is the most satisfactory method of selection – it is certainly the method adopted by almost all leading medical and scientific journals. Although other methods have from time-to-time been suggested, their disadvantages have always seemed to outweigh those of the established process.

Clearly, a journal such as the *British Journal of Cancer* could not operate without an immense amount of hard work and goodwill from a large number of anonymous, unpaid referees. One could perhaps ask the question why scientists are willing to take on the task. This is a pertinent question, both for relatively junior referees who are likely to want to spend every minute possible at the bench or in the clinic and for more senior people who are likely already to be burdened with administrative duties of various kinds. Probably the best answer is that being a referee gets one 'into the thick of things'. It allows a scientist to see new developments in a field 'as they happen', it allows him or her the opportunity to advise others as to how their work may be improved and it helps to prevent the publication of erroneous or misleading results. It could perhaps be argued that a scientist has a responsiblility to referee as many papers as the number of referees who will have reviewed his own work. This is probably too rigid a view, however, as different people contribute to the field in general in different ways. Certainly a reluctant referee is unlikely to be the best. It has been said that 'if one wants a job doing quickly and well, ask a busy person'. In line with this view, some of our quickest and best referees are people with enormous work loads. Since the Editorial Office of the *British Journal of Cancer* moved to Cambridge 2 years ago, Peter Twentyman has used a total of 850 referees, of whom 44 have reviewed between 10 and 19 papers, 13 have reviewed more than 20 and one has reviewed a grand total of 40. These figures do not include re-appraisals which we will discuss later. To all of them, our sincere gratitude.

The number of referees used for a particular paper will depend on a number of factors, but it will be either two or three. The advantages of using three referees are that it gives a degree of latitude such that, if one referee fails to respond, the Editor still has two reports and it also, usually, makes a third report available when the first two to arrive contain conflicting opinions. In either of these circumstances, to seek the opinion of an additional referee once a problem has arisen is bound to lead to delay in the decision process. The disadvantages are that the average referee will be asked to review 50% more papers and the amount of paperwork with which the Editorial office has to deal is increased by 50%. The arguments for using a third referee are likely to prevail when referees are geographically distant (and harder to chase for their reports), when they are being used for the first time, especially if they have been selected from reference lists rather than by personal contact (see below) and when the paper in question is quite outside the realm of expertise of the Editor or Clinical Editor. The different practices of Peter Twentyman (usually three referees) and Peter Selby (usually two) result from their own experience with different groups of referees in the different disciplines.

There are a number of ways by which referees may be selected by the Editor or Clinical Editor. The Editorial Board of the Journal, currently consisting of 48 eminent scientists and clinicians, who have accepted a role in the direction of the *British Journal of Cancer*, clearly constitutes a major resource with regard to selection of referees. Beyond this, undoubtedly the best method of selection relies on the person's track record as a referee for the Journal. 'Once a good referee, always a good referee' is likely to be true. In this context a 'good referee' is one who responds quickly and whose report is likely to be helpful to both author and Editor. The problem here of course is that the better a referee is, the more papers he is liable to be sent by various journals. One imagines that, somewhere along the line, a feedback loop operates and the referee's performance will deteriorate or he will start sending papers back unrefereed. For this reason and also because of a strong desire to avoid too much 'inbreeding' in which small groups of scientists are repeatedly reviewing each other's papers, we are constantly attempting to increase our panel of referees. Personal contact is the best way of doing this and regular attendance at cancer research meetings in the UK and abroad is an important part of an Editor's duties. Beware, there is no such thing as a free beer!

If personal knowledge of suitable referees for a paper fails, then the Editor can pursue a variety of options. Clearly the reference lists of submitted paper are likely to be valuable. However, it is important when using this route, to select only from references which have appeared during the previous 2 or 3 years and which relate directly to the question being addressed in the submitted paper. If an author is presenting data which conflict with previously published results, then the authors of earlier papers may be important potential referees although, of course, the Editor will wish to view the reports of such referees with particular circumspection. Problems can arise, when selecting referees from reference lists, if the author 'to whom reprint request should be addressed' is not the first author of a paper. This may indicate that the first author was a visitor or student who is likely to have moved on! The probability of receiving a report from a referee selected in this way is much lower than from a personal contact and the use of more than one such referee for a particular paper is generally avoided. Another valuable source of referees for 'difficult' papers are the abstract books of recent BACR/ACP or ASCO/AACR meetings. The latter are particularly useful because of the sheer volume and the presence of a subject-index. Perhaps the most difficult papers for which to find referees are those reporting results in rather obscure fields where all the quoted references are either from the present authors or are more than 5 years old. Particularly useful in this instance are a small number of 'generic referees' who are very experienced scientists able to take a broader view of the issues raised.

In theory it should be possible to assign key words to the list of referees held on our computer system. When the key words for a newly received paper are entered, the computer would then suggest referees and give details as to how many other papers they have been sent over the previous year. Computer programmes to do this are under development but the implementation of such a system presently remains an attractive option for the future.

One may ask the question 'In which circumstances would it be unethical for a referee to review a given paper?'. The simple answer is that no-one should referee a paper when he feels that his opinions are likely to be influenced by factors other than the merits of the paper before him. Clearly an author cannot review his own paper. It is also likely to be difficult for a referee to review a paper coming from the same Department or Institute. A more tricky situation arises for papers authored by the referee's former colleagues or students now working elsewhere. This will be a matter of individual judgement. Sometimes a referee may have previously seen a paper, having been asked by the author to take an informal look at it and give an opinion before submission. There is no reason why, having acted in this capacity, the same person cannot correctly act as an impartial referee. Likewise, the fact that a given person has provided a cell line or an antibody used in a particular piece or work is no reason why he should not subsequently review a paper describing the results of the work.

Once the referees have been selected, a copy of the manuscript is sent together with a letter requesting the referee to review the paper. The letter forms the top portion of a 'pro-forma', the lower part of which is the referees' report to the Editor. A second sheet is also sent upon which the referee can make specific remarks to the authors. We are currently using somewhat different forms in our two offices. Peter Selby uses a form developed by Michael Moore during his time as Editor (Figure 1) in which the remarks to the Editor consist only of a general grading and a blank space. Peter Twentyman, on the other hand, uses a form in which the referee is also asked to rate the paper in terms of a number of stated criteria (Figure 2). These criteria were taken (with permission!) from the form used by the *Journal of the National Cancer Institute*, with the addition of the final criterion 'originality and significance of results'. It is very interesting how referees deal with these forms. Virtually all referees will tick a general rating. However, of 100 recent referees who ticked categories 2 or 3 (i.e. minor or major revisions) only 61 ticked the boxes indicating whether or not they wanted to review the revised version (35 said 'yes' and 26 said 'no'). When a referee does not indicate a preference here, we assume that he is equally happy to see the paper again or not. Of these same 100 referees who used the revised form, 92 ticked a grade for each specific criterion, 5 ticked in only some categories and 3 did not use this part of the form at all. Nearly every referee writes some comments to the Editor and this can be anything from a single word to a lengthy exposition. We do not have a problem with this multiplicity of approach. It is nearly always possible to deduce the broad message which the referee wishes to convey.

The time taken for referees to respond is, expectedly, very variable but perhaps disappointingly long. For 100 recent referees, the mean time was 31 days. Of these 100 referees, only 23 reports were received with 3 weeks from dispatch and 23 failed to reply with 6 weeks, despite the application of 'chasing procedures' by the Editorial Office in some cases. More seriously, out of the 100 papers for which three referees were used, for only 50% had we received two or more reports by 30 days. In the light of these figures, our mean time from receipt of a paper to informing the author of the decision (53 days) may be put into context.

How could we improve these figures? One possibility would be the application of more rigorous 'chasing procedures'. There is a real danger, however, that over-zealous chasing will antagonise referees and make them reluctant to accept papers from the *British Journal Cancer*. Another possibility is to contact referees by telephone or FAX *before* sending a paper out, in order to ensure that they are not away and are agreeable to refereeing the paper. This is a costly and time-consuming option, but one which we are currently considering trying.

We could, of course, pay referees for their services. One of the new cancer journals is using this approach. Of course, the money would have to come from somewhere, either from authors or subscribers, certainly from the total pool of cancer research funds and to the cost of the actual payment would be added the cost of administering the scheme. The potential difficulties are enormous. Would, for instance, a referee be paid if his report was late? How would one deal with a three line report clearly dashed off in 5 minutes after a quick glance at the paper? We believe that most people see the refereeing of papers as part of the overall job of 'being a scientist'. The concept that a scientist's priorities within his overall job should be dictated by the various fees on offer is not one to which we would wish to subscribe. A perhaps more acceptable alternative is the giving to referees of 'reprint vouchers', a scheme recently adopted by the *International Journal of Radiation Biology*. Such vouchers can be used by referees to obtain an increased number of free reprints of any article which they themselves subsequently publish in the Journal. This is an attractive option which we are currently considering in detail.

To what extent do referees agree or disagree in their views of a particular paper? Again, looking at 100 papers, seen by either two or three referees, for 22 of them there were gross differences of opinion (either 'minor modification' alongside 'unacceptable' or 'acceptable without modification' alongside 'major changes required'). Where such a difference exists the Editor is likely to want to read the paper fully himself before re-reading the reports and reaching a decision. Only by experience does an Editor get to know his referees and be able to put their opinions in context. Some referees tend to be harsh in their judgements, nearly always rejecting or requiring major revisions, whilst others are much more easily satisfied. The Editor is also likely to be aware that a given referee is liable to assume a rather independent stance and this can, at times, be of particular value. One very important role that a referee may play, if particularly familiar with a subject, is in pointing out to the Editor that the contents of a paper have been, to a lesser or greater extent, previously published by the authors. Such a revelation, if correct, can present a major ethical problem which the Editor will wish to consider carefully before contacting the authors.

The most important thing for referees to bear in mind when making 'Remarks which may be communicated to authors', is that THEY SHOULD NOT INDICATE ON THIS SHEET WHAT ADVICE THEY HAVE GIVEN TO THE EDITOR REGARDING THE FATE OF THE PAPER. This is a matter between the referee and the Editor, a relationship to which the author is not a party. If a referee is making a number of specific suggestions to the authors for modifications it is very helpful if these can be numbered and divided into 'major scientific points' and 'minor changes'. The former may include suggestions for necessary additional work, the pointing out of major defects in the design or execution of the experiments and statements as to why the conclusions are not supported by the data. Minor points will include the correction of matters of fact, grammatical or typographical errors, pointing out of missing or incorrect references, errors in units, Tables or Figures, etc.

The amounts that referees write is extremely variable – from just a very few lines to four or five pages of typescript. Clearly a referee is likely to write less if he is recommending 'acceptance with minor modification' than if he is recommending 'major changes required' and asking to see the paper again following revision. It is a moot point whether or not a referee should spend a lot of time writing a detailed critique of a paper which he is recommending should be rejected. An author may feel that to be told 'This is old-fashioned science leading to a predictable and unimportant result' is not very constructive. On the other hand, if that is what the referee feels, what more is there to say?

One absolutely vital role that a referee can play is letting the Editor (and the authors) know when he considers that a paper is too long and/or contains too many Figures or Tables. Papers are submitted in which an interesting but well-defined set of results is followed by 10 or 12 pages of rambling discussion in which a large range of only loosely connected matters are dragged out for minute examination before being returned to the archives. Likewise, papers appear with 15 or 20 Figures showing sometimes the same data in five different ways or five individual replicate experiments with almost identical results. With the pressure on space which currently prevails, we are most anxious that only essential data are presented, they are only presented once and the discussion is confined to the immediate issues raised by the results presented. Authors should note that, when all three referees state that 'the discussion is far too long', a reduction from ten pages to nine in the revised version is unlikely to dispose the Editor kindly!

When making comments to the authors, it is essential that referees do so in a clear, unambiguous way. An author who simply cannot understand what a referee is saying does not have the option of telephoning him to find out and will therefore have to guess. When a revised manuscript is re-appraised by the referee and it turns out that the author has guessed wrongly, there is likely to be disatisfaction on both sides.

A referee who finds a paper to be full of grammatical and spelling mistakes is in a difficult position. This is, of course, more likely to be the case for papers submitted from countries where English is not the native language. Having said that, however, it is also true that the occasional paper from a major centre in the UK is submitted in quite unacceptable form, clearly not having been properly checked by the authors. If the referee finds the paper unintelligible, he should simply return it to the Editor and say so. If the problem is less severe, he may let the Editor know that 'the paper is badly written and will require considerable sub-editing' or he may attempt to list specific corrections. This can be done either in 'comments to the authors' or actually on the copy of the manuscript (preferably in pencil). The extent to which referees feel able to involve themselves to this degree will depend upon their time available. The efforts made by some referees are, however, notably beyond any reasonable expectations.

Most referees will be conscious of the fact that papers sent for review are 'privileged communications'. Their contents should not therefore be passed on in any way to third parties. The only exception to this would be a situation where a referee is asking a colleague to assist in the review – in this case the confidentiality condition will similarly bind the colleague. The referee should not relate the contents of the paper to his other colleagues or his students. This can be a very severe restraint indeed. Even more clearly, the contents of the reviewed paper cannot be referred to by the referee in his own papers and the referee should not use his privilege of seeing the authors' data in any way which will be either deleterious to the author or directly advantageous to himself.

Most papers which have undergone major revisions will be seen again by one or more of the referees. When papers are sent out for re-appraisal, no forms are sent with the request, but, in addition to the revised manuscript, the referee will usually receive copies of ALL the referees' reports on the original paper plus a copy of the authors' response letter. In this way, a referee is able, when judging the revised version, to assess the extent to which the other referees agreed with his original evaluation and the extent to which the author has successfully dealt with the various points raised. Many referees have commented that they find this particularly helpful.

In making a re-appraisal, the referee will hopefully not be too 'nit-picking'. Authors may often make changes in response to some points raised by referees but decline to make changes in response to others. The referee will have to decide which are the important 'sticking points' and which are less important 'matters of emphasis'. Only in rare cases is it legitimate for a referee to raise new issues in his re-appraisal unless of course these have resulted from the revisions made. In the end, following re-appraisal and occasionally after further consultation, the Editor will make a decision. In some cases this will mean that one of the referees will be overruled. This in no way means that the views of such a referee have been ignored, only that, on balance, they have not prevailed in this particular instance. Under these circumstance, the Editor may well write to the referee explaining what has happened.

We hope that in this article we have provided information which will help authors to understand what happens to their papers, why it takes so long, and how decisions are reached. We hope that new referees will find it useful to have at least a glimpse of the overall picture. If everyone filled in the forms in the same way or wrote their comments in the same style, the life of an Editor would be much less interesting. We are grateful to all our referees for their efforts. We know that authors often find the referees' criticisms helpful and contributory towards a better final product. It can, of course, be frustrating for an author to receive adverse comments from a referee, but where would we be without them?

Peter Twentyman

Cambridge

Peter Selby

Leeds

Figure 1

### BRITISH JOURNAL OF CANCER

Dr Peter Selby,
Institute for Cancer Studies
St. James's University Hospital
Beckett Street, Leeds LS9 7TF
Telephone: 0532 429883
Facsimile: 0532 429886

Cancer Research Campaign

(British Empire Cancer Campaign for Research)

1990

Dear

I should be grateful for your opinion on the enclosed paper, with particular reference to the information it presents being new, a distinct contribution to knowledge, and relevant to the problem of cancer.

If you cannot deal with this manuscript within a fortnight, please send it back by return post, or preferably pass it to a colleague with experience in the field.

Yours sincerely

**Peter Selby,** *Clinical Editor*

Details of paper

MS Number ......................

Title ..............................................................................................................................

..............................................................................................................................

Author ..............................................................................................................................

.............................................. Pages ................ Figures .................... Tables ............

Please tick appropriate places

1. Acceptable, without modification ..........
2. Acceptable with minor modification ..........
3. Major changes required ..........
   (a) revised version need not be re-referred ..........
   (b) revised version to be re-referred ..........
4. Unacceptable ..........

Brief comment

Signature of Referee

Figure 2

### BRITISH JOURNAL OF CANCER

Editorial Office
MRC Clinical Oncology and Radiotherapeutics Unit
Hills Road
Cambridge CB2 2QH
Tel: 0223 210115

**Cancer Research Campaign**

2 Carlton House Terrace, London SW1Y 5AR
Tel 071-930 8972

| Manuscript Number |
|---|
| Title and Authors |

Dear

I should be grateful for your opinion on the enclosed paper, with particular reference to the information it presents being new, a distinct contribution to knowledge, and relevant to the problem of cancer.

If you cannot deal with this manuscript within a fortnight, please send it back by return post, or preferably pass it to a colleague with experience in the field.

Yours sincerely

Peter Twentyman, Ph.D., Editor

### Reviewer's Summary Comments to the Editor

Please indicate your final recommendation (Tick One)

- ☐ 1. Acceptable, without modification
- ☐ 2. Acceptable with minor modification
- ☐ 3. Major changes required
- ☐ 4. Unacceptable

If you recommend 2 or 3, revised version to be re-refereed? ☐ YES ☐ NO

Please evaluate this manuscript using the following criterion: A-Outstanding; B-Very good; C-Good; D-Fair; and E-Poor

| | | | | | |
|---|---|---|---|---|---|
| Importance of the Question Addressed | A | B | C | D | E |
| Study Design | A | B | C | D | E |
| Study Execution | A | B | C | D | E |
| Data Analysis | A | B | C | D | E |
| Clarity of Presentation | A | B | C | D | E |
| Originality and significance of results | A | B | C | D | E |

(Circle)

Use this space for additional comments to the Editor (Please type or print clearly)

PLEASE USE PAGE 2 OF THIS FORM FOR ALL COMMENTS TO THE AUTHOR. THESE SHOULD INDICATE CLEARLY THE REASONS FOR YOUR RECOMMENDATION TO THE EDITOR.

Signature........................................ Date........................................ Phone Number ........................................